# Unraveling the Puzzle: A Case Report Questioning the Causal Relationship Between Subarachnoid Hemorrhage and Microscopic Polyangiitis

**DOI:** 10.7759/cureus.41088

**Published:** 2023-06-28

**Authors:** Ashbina Pokharel, Indira Acharya, Joseph Skender

**Affiliations:** 1 Internal Medicine, Beaumont Hospital, Royal Oak, USA; 2 Internal Medicine, MedStar Union Memorial Hospital, Baltimore, USA; 3 Rheumatology, Beaumont Hospital, Royal Oak, USA

**Keywords:** anca-associated vasculitis, microscopic polyangiitis, cerebral vasculitis, vasculitis, subarachnoid hemorrhage

## Abstract

Antineutrophil cytoplasmic antibody (ANCA)-associated vasculitides (AAV) are small-to-medium-vessel vasculitis, which includes granulomatosis with polyangiitis, microscopic polyangiitis (MPA), and eosinophilic granulomatosis with polyangiitis. MPA predominantly affects the kidneys and lungs. Subarachnoid hemorrhage (SAH), a life-threatening condition, rarely occurs with AAV. In this case, we present a 67-year-old female who presented with a sudden-onset headache after a recent diagnosis of ANCA-associated renal vasculitis. Kidney biopsy revealed pauci-immune glomerulonephritis, and serum was positive for ANCA along with myeloperoxidase antibody. A computed tomography scan of the head revealed both SAH and intraparenchymal hemorrhage. The patient was managed medically for SAH and intraparenchymal hemorrhage. ANCA vasculitis was treated with steroids and rituximab, and the patient showed improvement.

## Introduction

Microscopic polyangiitis (MPA) is a type of antineutrophil cytoplasmic antibody (ANCA)-associated vasculitides (AAV) defined as pauci-immune necrotizing vasculitis of small vessels. Though the exact cause of AAV is not fully understood, the pathogenesis of AAV includes a complex process of immune dysregulation of genetics and environmental factors. The lungs, kidneys, and skin are commonly affected organs, and neurological manifestation has rarely been reported. Mononeuritis multiplex is the most common neurological manifestation of AAV. Stroke, hypertrophic pachymeningitis, massive intracerebral hemorrhage, subarachnoid hemorrhage (SAH), and spinal SAH associated with MPA have been described in case reports [1]. However, per the literature review, it is unclear if MPA has a causal relationship with SAH. Here, we report a rare case of SAH and intraparenchymal hemorrhage concurrent with MPA.

## Case presentation

A 67-year-old female with a recent diagnosis of ANCA vasculitis and past medical history of hypothyroidism, hyperlipidemia, and anxiety presented to our emergency department with a diffuse headache and pressure-like sensation for two hours. Eight days before the presentation, she presented to her primary care physician for fatigue and poor appetite ongoing for six months. She also reported weight loss of about 40 pounds and intermittent low-grade fever for one week. Further lab work revealed a significant increase in creatinine to 2.36 mg/dL (reference range: 0.50-1.1 mg/dL) compared to 0.8 mg/dL two months prior. As a result, she was sent to the emergency room for further workup.

During this hospitalization, the infectious workup, including blood culture, was negative. The immunological workup revealed a positive ANCA titer of 1:80 (normal: <1:20) along with an elevated myeloperoxidase antibody level of 4.6 U/mL (reference: <3.5 U/mL) and elevated C-reactive protein level of 155 mg/dL (normal: <8 mg/dL). The left kidney biopsy revealed findings consistent with ANCA-associated necrotizing crescentic glomerulonephritis (pauci-immune type). As a result, she was treated with intravenous methylprednisolone at a dose of 1 g per day for three days, followed by oral prednisone at a daily dose of 60 mg. Additionally, she received a single dose of 1 g rituximab and was discharged home after nine days of hospitalization. At the time of discharge, she was prescribed prednisone at a daily dose of 40 mg and trimethoprim-sulfamethoxazole for prophylaxis against *Pneumocystis jirovecii* pneumonia. Her serum creatinine at discharge showed improvement at 1.79 mg/dL.

Two days after discharge, she presented to the hospital with a headache for two hours. She described her headache as pressure-like, 7/10 in intensity, started on the occipital area, radiating to her lower neck, and not relieved by acetaminophen. Her vital signs at presentation were blood pressure of 170/68 mmHg, heart rate of 67 beats per minute, respiratory rate of 20 breaths per minute, temperature of 36.6°C, and oxygen saturation of 100% on room air. The laboratory results at the time of presentation are shown in Table 1.

**Table 1:** Laboratory results at presentation.

Labs	Results	Reference range
White blood cell count	12.6 bil/L	3.3–10.7 bil/L
Hemoglobin	10.2 g/dL	12.1–15.0 g/dL
Platelets	406 bil/L	150–400 bil/L
Sodium	132 mmol/L	135–145 mmol/L
Potassium	4.7 mmol/L	3.5–5.2 mmol/L
Bicarbonate	19 mmol/L	20–29 mmol/L
Creatinine	1.8 mg/dL	0.50–1.10 mg/dL
Protime	10.8 seconds	9.2–13.5 seconds

**Figure 1:**
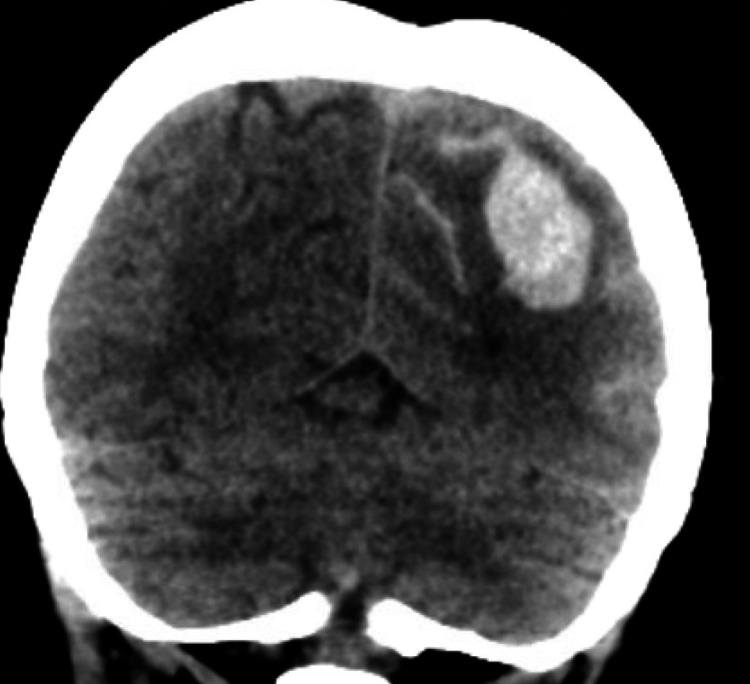
Computed tomography of the head demonstrating diffuse left-sided subarachnoid hemorrhage extending to the left ambient cistern, along with a 3.2 × 1.8 cm intraparenchymal hemorrhage at the left posterosuperior parietal lobe without midline shift.

**Figure 2:**
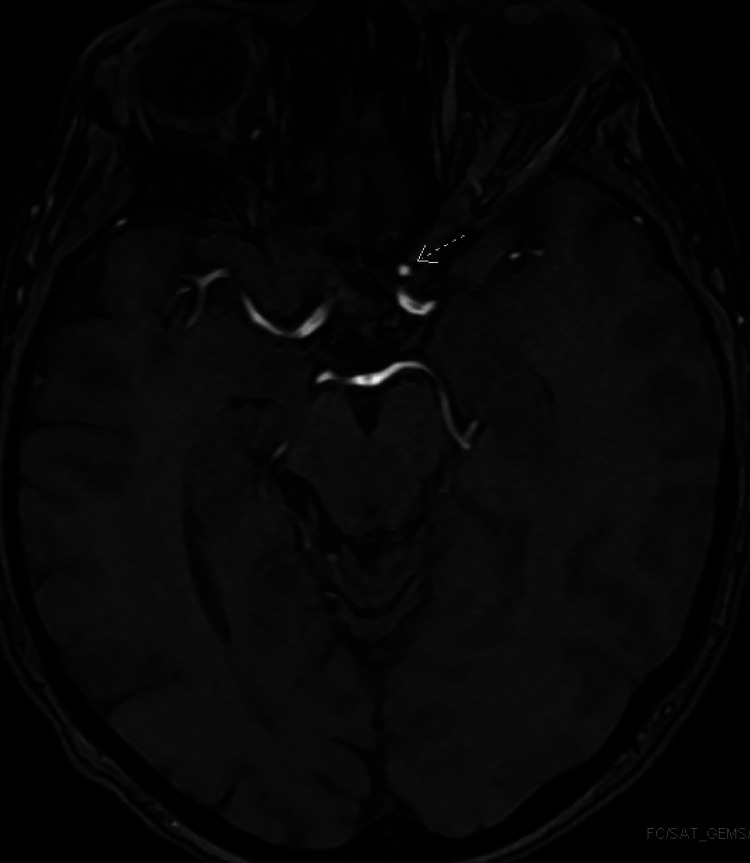
Magnetic resonance angiogram of the brain demonstrating a saccular aneurysm measuring 3 × 3 mm involving the paraclinoid segment of the left internal carotid artery (arrow).

On physical examination, significant findings included 4/5 strength in bilateral lower extremities, nuchal rigidity, and decreased sensation on the right side of the face. A computed tomography (CT) scan of the head revealed a diffuse left-sided SAH extending to the left ambient cistern, along with a 3.2 × 1.8 cm intraparenchymal hemorrhage at the left posterosuperior parietal lobe without midline shift, as shown in Figure 1. Her Hunt/Hess scale for SAH was grade II. Neurosurgery evaluated the patient and no acute surgical intervention was planned. She was admitted to the medical intensive care unit (ICU) for frequent neurological checks and closer hemodynamic monitoring, and the blood pressure was controlled with a nicardipine drip. A repeat CT of the head was performed 12 hours after the initial scan, which showed stable SAH as the prior CT. A magnetic resonance angiogram (MRA) of the brain revealed a saccular aneurysm measuring 3 × 3 mm involving the paraclinoid segment of the left internal carotid artery, as depicted in Figure 2. A cerebral angiogram was performed, which corroborated the findings of MRA but did not reveal any arteriovenous malformation or fistula. The angiogram also did not show any findings suggestive of vasculitis. Therefore, no neurosurgical intervention was performed. Nicardipine drip was weaned, and her blood pressure remained well controlled on oral medications. After four days, she was transferred out of the ICU to the progressive floor. During this hospitalization, she received a second dose of rituximab, 1 g, 14 days after the first dose. Following one week of hospital rehabilitation, her lower extremities and facial weakness improved, and she was discharged home on a prednisone dose of 40 mg daily.

## Discussion

AAV is associated with the presence of antibodies that target neutrophil antigens, primarily leukocyte proteinase 3 (PR3) and myeloperoxidase (MPO). The exact etiology and pathogenesis of AAV are complex and multifactorial, involving contributions from genetic factors, environmental components, infections, and immunological factors. The precise mechanism underlying cerebral aneurysm formation in MPA is not fully understood. It may be associated with arteritis of small and medium-sized vessels or necrotizing changes [2]. PR3-ANCA and MPO-ANCA have high sensitivity and specificity for AAV, but they can also be present in cases of inflammation, infection, or drug-induced conditions. A positive biopsy supports the diagnosis of vasculitis and is recommended to obtain [3]. In our case, the diagnosis was supported by immunology, kidney biopsy, and clinical manifestations. The clinical manifestations of AAV vary depending on the affected vascular bed. While the lungs and kidneys are the most commonly involved organs, AAV can also affect the upper respiratory tract, skin, heart, and nervous system.

The most common neurological manifestations of AAV include mononeuritis multiplex and hearing loss. A sural nerve biopsy revealed necrotizing vasculitis in 80% of affected patients, and acute axonopathy was seen in nerve conduction studies [4]. Overall, 17-30% of neurological involvement in cases of AAV included the central nervous system, and the most common manifestations included cerebral hemorrhage, pachymeningitis, and non-hemorrhagic cerebral infarctions [4]. These events typically occurred within the first 10 days of admission [2]. However, in the case report by Iyoda et al., cerebral infarction was documented to occur between days 2 and 26 of admission [5], and in the case report by Ku et al., multiple cerebral infarctions were observed after 25 days of admission [6]. In our case, SAH was diagnosed within 11 days of the initial presentation. SAH, regardless of treatment, is associated with a mortality rate of over 50%. Multiple case reports have demonstrated a poor prognosis in patients with MPA and cerebrovascular accidents [2]. This could be attributed to the inherently poor prognosis of cerebrovascular disease, which is further complicated by the use of immunosuppressive therapy for AAV. However, in our case, successful management was achieved through medical intervention, resulting in a stable condition at the time of discharge.

The management of AAV typically involves the use of immunosuppressive medications. For non-organ or non-life-threatening active granulomatosis with polyangiitis (GPA)/MPA, the induction of remission is often achieved through the combination of rituximab (or methotrexate and mycophenolic acid) and glucocorticoids, which are gradually tapered over several months, or avacopan [3]. Whereas in cases of the organ or life-threatening active GPA/MPA, it is recommended to start rituximab or cyclophosphamide in combination with glucocorticoids tapered over months or avacopan [3]. Plasma exchange is recommended in cases of rapidly progressive glomerulonephritis. For the maintenance of remission, it is recommended to continue or switch to rituximab (or azathioprine or methotrexate) over 24-48 months, along with tapering of glucocorticoids, or discontinuation of avacopan [3]. In our case, the patient was managed with rituximab and steroids.

## Conclusions

The occurrence of cerebrovascular disease simultaneously with MPA is rare. Clinicians should be aware of the potential risk of SAH and other cerebrovascular manifestations in newly diagnosed AAV patients. Numerous reported cases have indicated a poor prognosis in such situations. Hence, physicians should exercise heightened vigilance regarding the presence of cerebral aneurysms in individuals with MPA, particularly if the patient presents with neurologic symptoms. Further studies are necessary to establish a clearer correlation between MPA and SAH or other cerebrovascular events, as well as to explore additional neurological manifestations associated with the condition.
